# EpCAM Expression in Lymph Node Metastases of Urothelial Cell Carcinoma of the Bladder: A Pilot Study

**DOI:** 10.3390/ijms18081802

**Published:** 2017-08-18

**Authors:** Christa A. M. van der Fels, Stefano Rosati, Igle J. de Jong

**Affiliations:** 1 Department of Urology, University Medical Center Groningen, University of Groningen, 9700 RB Groningen, The Netherlands; i.j.de.jong@umcg.nl; 2 Department of Pathology, University Medical Center Groningen, University of Groningen, 9700 RB Groningen, The Netherlands; s.rosati@umcg.nl

**Keywords:** urothelial cell carcinoma, immunohistochemistry, lymph node metastases, EpCAM

## Abstract

In this retrospective pilot study, the feasibility of the epithelial cell adhesion molecule (EpCAM) as an imaging target for lymph node (LN) metastatic disease of urothelial cell carcinoma (UCC) of the bladder was investigated. LN metastases and LNs without metastases of patients who underwent pelvic lymph node dissection because of muscle invasive bladder cancer (MIBC) were used. Primary tumors of the same patients were used from cystectomy specimen, transurethral resections, and biopsies. A pathologist, blinded to clinical data, scored EpCAM immunoreactivity. This method determines a total immunostaining score, which is the product of a proportion score and an intensity score. EpCAM expression was observed in 19/20 (95%) LNs with UCC metastases and in 11/12 (92%) of the primary tumors. EpCAM expression was absent in 14/14 (100%) LNs without metastases. Median EpCAM expression (TIS) in LN metastases was 5 (IQR 2.0–8.0) and in the primary tumors 6 (IQR 2.3–11.0). Based on the absence of staining in LNs without metastases, EpCAM show high tumor distinctiveness. EpCAM seems to be a feasible imaging target in LN metastases of UCC of the bladder. Pre- and perioperative visualization of these metastases will improve disease staging and improve the complete resection of LN metastases in MIBC.

## 1. Introduction

Imaging is needed to determine local extension and lymph node status of muscle invasive bladder cancer (MIBC) [1]. C-Choline PET/CT have been proved to be more sensitive to assess the presence of lymph node metastases in patients with bladder cancer, compared to contrast-enhanced CT. However, it still lacks sensitivity (10–59%) and specificity (64–90%) in diagnostic information in preoperative nodal staging of patients with invasive BC [2,3]. To date, FDG PET/CT seems to be the best performing method to detect lymph node metastases, with the highest sensitivity and specificity compared to other imaging modalities [4,5]. However, lymph node metastatic lesions less than 1 cm can still not be detect by this method [4]. In addition, the change in management is relatively small [1,6]. Pelvic lymph node dissection remains the gold standard for nodal staging.

New diagnostic imaging modalities that can assess lymph node metastases of MIBC more properly than the available current modalities are preferred. Improving the identification of lymph node metastases will give us a more reliable staging in disease management. Besides, improving the identification of lymph node metastases perioperative will be helpful to improve the number of lymph nodes removed. Retrospective studies previously suggested that increasing lymph node retrieval may have a direct therapeutic effect either by more properly assigning a pathological category or by truly removing micrometastatic disease. Increasing nodal counts has an association with improved outcomes after radical cystectomy, specifically in those with node-positive disease [7,8,9].

By adding a specific target to molecular imaging modalities, tumor specific imaging modalities could be obtained. Previous target selection criteria scoring systems criticized multiple proteins as potential biomarkers for tumor targeted imaging techniques [10]. Because of its characteristics that resulted in a high total score, EpCAM seems a suitable candidate for these purposes [10]. Epithelial cell adhesion molecule (EpCAM; syn. GA733-2, TACSTD1, KSA, EGP40, CD326, 17-1A, HEA125, MK-1, EGP-2, EGP-34, ESA, KS1/4), is a transmembrane glycoprotein expressed exclusively in normal epithelial tissues and epithelial-derived neoplasms [11]. Immunohistochemical studies revealed that EpCAM is overexpressed on various carcinoma cells in breast, prostate, ovarian, lung, colon, renal, and gastric cancer [12]. The expression rates of EpCAM in urothelial cell carcinoma (UCC) of the bladder varies between 27% and 99% in different studies. Besides, EpCAM expression has also been found in normal urothelial cells [11,13,14]. Because of the wide variance of EpCAM expression in the primary tumor, the expression of EpCAM in lymph node metastases of urothelial cell bladder cancer has not been determined yet, as far as we know. Previously, EpCAM expression was highly found in lymph node metastases of prostate cancer [15,16] and lymph node metastases of urothelial carcinomas of the renal pelvis [17].

In the current study, the expression of EpCAM in lymph node metastases of UCC of the bladder was compared to the expression of EpCAM in matched lymph nodes without metastases and to the expression of EpCAM in the matched invasive primary bladder tumor.

## 2. Results

### 2.1. Immunoreactivity

Lymph node metastases and primary bladder tumor samples showed the typical histologic aspect of UCC, without differentiation of other tumor types. After immunohistochemistry, all 34 lymph nodes were available for analyses. Two (2 out of 14) of the primary tumor samples (patient 4: transurethral biopsy and patient 10: cystectomy specimen, Table 1) only showed CIS and pTa and no invasive tumor. Since invasive tumor is needed for a proper comparison between primary tumor and matched lymph nodes, these samples were not suitable for evaluation. Scoring of EpCAM immunoreactivity per lymph node and primary tumor sample is presented in Table 1.

EpCAM expression was observed in 19 out of 20 (95%) lymph nodes with UCC metastases. Median EpCAM expression (TIS) in lymph node metastases was 5.0 (inter quartile range: 2.0–8.0). Median proportion score was 3.0 (inter quartile range: 2.0–4.0) and median intensity score was 1.5 (inter quartile range: 1.0–2.0). If PS was low (1–2), IS was low too (1). Strong intensity score was only seen in two lymph nodes. EpCAM expression was absent in 14 out of 14 (100%) lymph nodes without metastases.

EpCAM expression was observed in 11 out of 12 (92%) primary UCC of the bladder. Median EpCAM expression (TIS) in primary tumor was 6.0 (inter quartile range 2.3–11.0). Median proportion score was 3.0 (inter quartile range: 1.5–4.0) and median intensity score was 2.0 (inter quartile range: 1.0–2.8). If PS was low (1–2), IS was low too (1).

### 2.2. Staining Pattern

**Figure 1:**
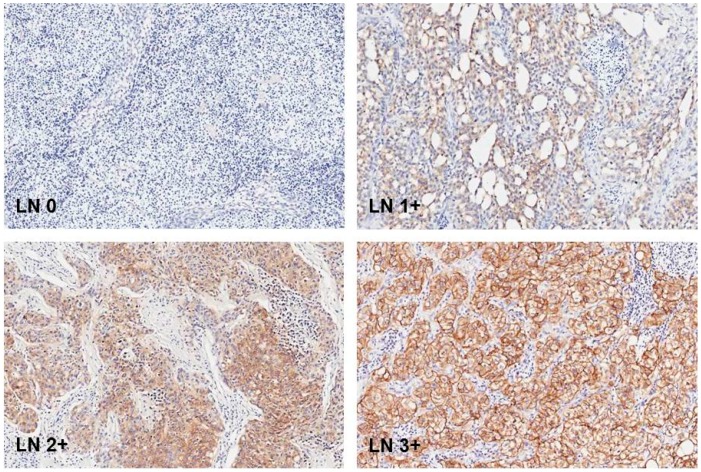
Normal lymph node without EpCAM expression and lymph node metastases with EpCAM expression with intensity score **1**: weak; **2**: moderate; and **3**: strong. Original magnification: 200×.

**Figure 2:**
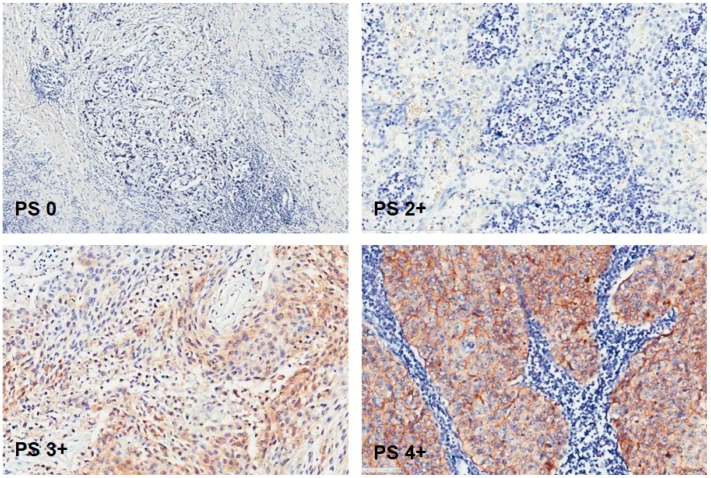
Lymph node metastasis without EpCAM expression. Lymph node metastases with EpCAM expression with proportion score **2**: 10–50%; **3**: 51–80%; and **4**: >80%. Original magnification: 200×.

**Figure 3:**
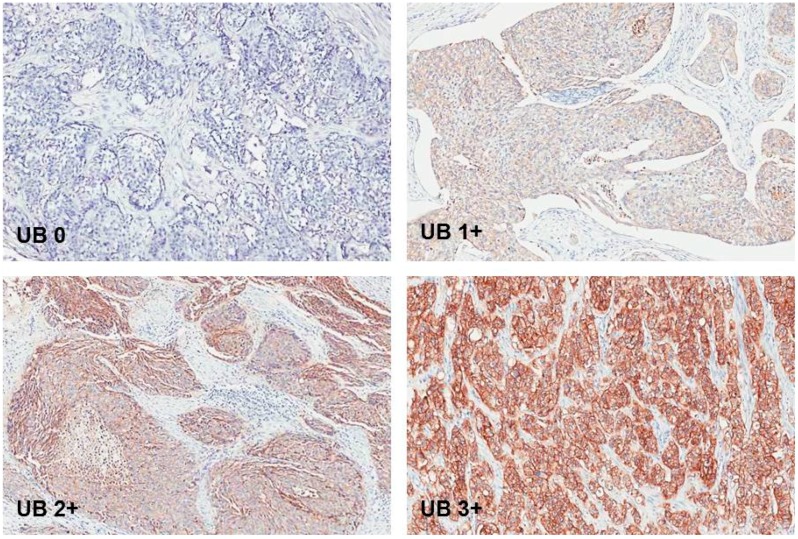
Primary tumor in urinary bladder without EpCAM expression and with EpCAM expression with intensity score **1**: weak; **2**: moderate; and **3**: strong. Original magnification: 200×.

EpCAM brown staining was seen membranous in UCC cells of lymph nodes. Figure 1. Only one lymph node with UCC metastasis did not show EpCAM expression at all. The rest of the lymph node metastases showed EpCAM brown staining in different proportion scores. Figure 2. EpCAM brown staining was also seen membranous in UCC cells in primary tumor samples. Figure 3.

## 3. Discussion

The current study shows a high EpCAM expression in lymph node metastases of UCC of the bladder (19 out of 20), and absent of staining for EpCAM in lymph nodes without metastases (0 out of 14). However, there was a variance in the proportion and intensity of the expression amongst different lymph nodes. Previously, “overexpression” of EpCAM was defined as a TIS > 4 [11,18]. In addition, four subgroups of overexpression have been defined: TIS 0, no expression; TIS 1–4, weak expression; TIS 6 and 8, moderate expression; TIS 9 and 12, intense expression [11,19]. According to these defined subgroups, from lymph node metastases; 1 (5%) did not show expression; 9 (45%) showed only weak expression; 6 (30%) showed moderate expression; and 4 (20%) showed intense expression in the current study. However, these subgroups were defined in order to evaluate the predictive value of EpCAM expression on survival in clinical trials and to predict therapy response in patients treated with EpCAM-specific targeting agents [18,19,20]. About the diagnostic and prognostic value of EpCAM expression in UCC of the bladder, Bryan et al. showed that elevated urinary EpCAM was an independent prognostic factor for bladder cancer survival [21]. Brunner et al. also previously showed that EpCAM expression is associated with advanced stage, high grade and poor survival in UCC of the bladder [17]. In our study, the feasibility of EpCAM as an imaging target was determined and not for risk stratification. Due to the low number of patients (*n* = 14), the power of the statistics would be too weak to correlate staining intensity with clinicopathological parameters and outcomes after surgery. Another limitation of our study is that immunoreactivity was only scored by one pathologist.

EpCAM expression in lymph nodes was compared to the expression of EpCAM in the matched primary bladder tumor. All but one (11 out of 12) of the primary tumors showed EpCAM expression, however, also with variable proportion and intensity scores. TIS in the primary tumor was not always the same as TIS in the matched lymph node metastasis of the same patient. Two lymph node metastases of one patient showed TIS in the same subgroup, except for the lymph node that did not show expression at all. Spizzo et al. observed EpCAM negativity (TIS 0) in 56% of urothelial carcinomas and an EpCAM overexpression rate (TIS > 4) of 27% [11]. This is a major difference with the expression that was found in our primary tumors. More data have to be collected and our results should also be confirmed by using a second primary antibody. On the other hand, Spizzo et al. did not describe whether their UCCs were invasive or non-invasive. All evaluated tumors in our study were invasive, which could explain the differences of EpCAM expression in the primary tumor as well. Other studies showed a strong, circumferential membrane reaction of EpCAM in carcinoma cells of the urinary bladder. However, EpCAM expression was also seen on normal urothelial tissue in these studies. EpCAM expression on normal urothelial tissue was described as limited to the basal layers of the bladder and was not seen in superficial umbrella cells. Lymph node specimens of UCCs were not available [14,22]. In our study, strong positive staining of EpCAM in some parts of normal urothelial cells of the bladder was seen in basal layers, but also weak staining was seen in umbrella cells. Therefore, EpCAM does not seem suitable as a diagnostic target for primary UCC of the bladder.

As far as we know, the current study is the first that assessed EpCAM expression in lymph node metastases of UCC of the bladder compared to matched normal lymph nodes and the matched primary tumor. EpCAM showed a high sensitivity (95%) and specificity (100%) in lymph nodes and also a high sensitivity (92%) in the primary bladder tumor. Antibodies against EpCAM have previously been used to visualize micrometastases in lymph nodes of papillary thyroid cancer and non-small-cell lung cancer by immunohistochemistry. These lymph nodes were found to be free of metastases at routine histopathological examination, [23,24] showing the use of antibodies against EpCAM was highly sensitive in the detection of lymph node metastases. Previous studies also proved that antibodies against EpCAM do not react with lymphoid tissue [25,26]. The absence of EpCAM overexpression in normal lymph nodes supports the use of EpCAM as a target for bladder cancer lymph node metastases. Antigen-based targeted imaging could be useful to rule out (micro)metastases prior to radical cystectomy; or to use perioperatively to remove these metastases more properly.

## 4. Materials and Methods

### 4.1. Patient Samples

Samples of primary invasive UCC of the bladder, lymph node metastases of UCC of the bladder and matched lymph nodes without metastases of the same patients were retrieved from the archives of the Department of Pathology of University Medical Center, Groningen. Only lymph nodes with an extensive amount of metastases were useful. Formalin-fixed, paraffin-embedded blocks of small lymph nodes with only micro metastases were not suitable to cut into 4 mm thick sections for the use of immunohistochemistry. A total 20 lymph node metastases of 14 patients who underwent pelvic lymph node dissection because of MIBC were available as well as lymph nodes without metastases of the same patients (*n* = 14). Primary tumors of these patients were taken from cystectomy specimen (*n* = 7), transurethral resection of the bladder tumor (*n* = 6) and transurethral biopsy of the bladder tumor (*n* = 1). All tissue specimens were anonymously coded. According to Dutch law, no further Institutional Review Board approval was required (http://www.federa.org/). Trial registration number (UMCG Research Register): 201600084 (date registered: 02/04/2016).

### 4.2. Experimental Setup

EpCAM expression was determined by immunohistochemistry. Normal colon was used as the positive control and omission of the primary antibody on normal colon samples served as a negative control. After deparaffinization with xylene baths and decreasing grades of alcohol, antigen retrieval was performed by incubation with 0.1% protease for 30 min at room temperature. Endogeneous peroxidase was blocked with 0.3% hydrogen peroxide in PBS for 20 min in dark. Slides were incubated with primary mouse monoclonal AB anti-EpCAM (clone VU-1D9, Leica Biosystems, Newcastle, UK), and diluted at 1:100 in 1% BSA/PBS for 1 h at room temperature. In the secondary step, slides were incubated with rabbit anti-mouse AB conjugated to polymer-horseradish peroxidase (DAKO, Glostrup, Denmark), and diluted at 1:100 in 1% BSA/PBS with 1% AB serum. In the tertiary step, goat anti-rabbit AB conjugated to polymer-horseradish peroxidase (DAKO, Glostrup, Denmark) was used, and diluted at 1:100 in 1% BSA/PBS with 1% AB serum. Secondary and tertiary antibodies were incubated for 30 min at room temperature. After every step, slides were washed with PBS and dried. Next, slides were immersed for 10 min in a solution of 0.05% 3,3′-diaminobenzidine (Sigma-Aldrich, Steinheim, Germany) and 0.03% hydrogen peroxide in PBS in dark for visualization of the signal as brown staining. After washing with demineralized water, slides were slightly counterstained with hematoxylin, dehydrated by increasing grades of alcohol and when dried, mounted with Tissue Tek film (Sakura Finetek, Leiden, The Netherlands).

### 4.3. Assessment of Staining Patterns

All samples were scored in total for EpCAM immunoreactivity by a pathologist (SR) blinded to clinical and pathological data, according to a previous established method [11]. This method consisted of a Proportion Score (PS) and an Intensity Score (IS), together resulting in a Total Immunostaining Score (TIS). The PS represents the estimated amount of positively stained cells (0, none; 1, <10%, 2, 10–50%; 3, 51–80%; 4, >80%). The IS describes the estimated staining intensity (0, no staining; 1, weak; 2, moderate; 3, strong). Specimens in which one or more tumor areas with different staining intensities were present were scored for the most prevalent intensity. The TIS (TIS = PS × IS) ranges from 0 to 12 with 9 possible values (0, 1, 2, 3, 4, 6, 8, 9, and 12) [11].

### 4.4. Data Analysis

Descriptive analyses were used to describe the results and are shown as median score. SPSS statistics (version 23.0 for Windows, IBM Corp., Armonk, NY, USA) was used for analyses.

## 5. Conclusions

In conclusion, EpCAM shows high tumor distinctiveness, because of the absence of staining in LNs without metastases. Based on this study, EpCAM could be used as an imaging target for bladder cancer lymph node metastases. Pre- and perioperative visualization of these metastases will improve disease staging and improve the complete resection of LN metastases in MIBC. Prospective clinical trials are needed to confirm the current results.

## Figures and Tables

**Table 1:** Scoring EpCAM immunoreactivity.

Patient	Age (Years)	Sex	Stage	LN Excised	LN+	Tissue Type	PS	IS	TIS
1	39	M	T2	2	1	Primary bladder tumor	3	2	6
						Lymph node normal	0	0	0
						Lymph node metastasis	3	1	3
2	77	M	T2	15	3	Primary bladder tumor	4	1	4
						Lymph node normal	0	0	0
						Lymph node metastasis	4	2	8
						Lymph node metastasis	3	2	6
3	79	F	T2	17	1	Primary bladder tumor	4	3	12
						Lymph node normal	0	0	0
						Lymph node metastasis	4	3	12
4	78	M	Tis	5	1	Primary bladder tumor	-	-	-
						Lymph node normal	0	0	0
						Lymph node metastasis	4	3	12
5	69	M	T2	16	2	Primary bladder tumor	0	0	0
						Lymph node normal	0	0	0
						Lymph node metastasis	1	1	1
6	55	M	T2	3	1	Primary bladder tumor	4	3	12
						Lymph node normal	3	2	6
						Lymph node metastasis	0	0	0
7	67	M	T2	8	4	Primary bladder tumor	3	1	3
						Lymph node normal	0	0	0
						Lymph node metastasis	2	1	2
						Lymph node metastasis	4	1	4
8	61	F	T2	8	3	Primary bladder tumor	4	2	8
						Lymph node normal	0	0	0
						Lymph node metastasis	4	2	8
						Lymph node metastasis	4	1	4
9	43	F	T2	22	5	Primary bladder tumor	1	1	1
						Lymph node normal	0	0	0
						Lymph node metastasis	2	1	2
10	68	M	T2	15	3	Primary bladder tumor	-	-	-
						Lymph node normal	0	0	0
						Lymph node metastasis	2	1	2
						Lymph node metastasis	2	1	2
11	64	F	T2	12	3	Primary bladder tumor	1	2	2
						Lymph node normal	0	0	0
						Lymph node metastasis	1	1	1
						Lymph node metastasis	0	0	0
12	64	M	T2	16	2	Primary bladder tumor	3	2	6
						Lymph node normal	0	0	0
						Lymph node metastasis	4	2	8
13	48	F	T2	7	1	Primary bladder tumor	4	3	12
						Lymph node normal	0	0	0
						Lymph node metastasis	3	2	6
14	78	M	T2	9	4	Primary bladder tumor	3	2	6
						Lymph node normal	0	0	0
						Lymph node metastasis	3	2	6
						Lymph node metastasis	4	2	8

Stage: T stage primary tumor, PS: Proportion Score, IS: Intensity Score, TIS: Total Immunostaining Score.

## Abbreviations

MIBC	Muscle invasive bladder cancer
LN	Lymph node
TIS	Total immunostaining score
PS	Proportion score
IS	Intensity score
UCC	Urothelial cell carcinoma
PBS	Phosphate buffered saline
BSA/PBS	Bovine serum albumin/phosphate buffered saline
AB	Antibody
EpCAM	Epithelial cell adhesion molecule
